# Single‐port robot‐assisted ureterolysis for retroperitoneal fibrosis: A less invasive path to functional recovery

**DOI:** 10.1002/bco2.70218

**Published:** 2026-05-04

**Authors:** Filippo Carletti, Alexandru Turcan, Flavia Tamborino, Luca Alfredo Morgantini, Lorenzo Salvodirocco, Marwan Alkassis, Fabrizio Dal Moro, Simone Crivellaro

**Affiliations:** ^1^ Department of Urology University of Illinois at Chicago Chicago Illinois USA; ^2^ Urology Clinic, Department of Surgery, Oncology and Gastroenterology University of Padua Padua Italy

**Keywords:** omental wrap, outcomes, peritoneal flap, same day discharge, single port, supine, ureterolysis

## Abstract

**Objective:**

The objective of this study is to evaluate the outcomes of an initial series of single‐port robot‐assisted ureterolysis (SP‐RAU) in patients with retroperitoneal fibrosis (RPF).

**Patients and Methods:**

We prospectively collected surgical and clinical data from all consecutive patients undergoing SP‐RAU at our institution between April 2016 and May 2024. The primary endpoint was the achievement of a stent‐free status at 12 months.

**Results:**

Overall, 16 patients (21 renal units) underwent SP‐RAU with a minimum follow‐up of 12 months. All five patients with bilateral disease were managed with a staged approach in two separate surgical sessions. RPF was idiopathic in 37% and secondary in 62% of cases. Four procedures (19%) were aborted due to extensive fibrosis or high vascular risk (one unilateral right, two unilateral left and one second‐stage left unit). These patients had significantly longer preoperative stent duration (60 vs. 9 months; *p* = 0.006). Among the 17 completed procedures, no open conversions, intraoperative complications or transfusions occurred. Median operative time was 215 min (IQR 195–240), and median blood loss was 10 ml (IQR 10–20). Median length of stay was 11.5 h (IQR 7.8–26), with 52% of patients discharged the same day. At a median follow‐up of 25 months (IQR 18–50), complete symptom resolution was achieved in 82% of renal units, whereas 64% remained stent‐free at 12 months. The main limitations are the small sample size, single‐surgeon experience and absence of a comparator arm.

**Conclusion:**

SP‐RAU is a safe and feasible minimally invasive option for managing RPF. It achieves good functional success with minimal morbidity and enables same‐day discharge in over half of patients. Larger multicentre studies with longer follow‐up are needed to confirm these findings and establish the role of SP‐RAU in the management of RPF.

## 1 INTRODUCTION

Retroperitoneal fibrosis (RPF) is a rare condition characterized by chronic inflammation and fibrosis in the retroperitoneum, which can lead to the extrinsic compression of retroperitoneal structures such as the aorta, vena cava and, in the later stages, the ureters. Idiopathic RPF accounts for nearly 70% of all cases, and it is hypothesized to be a manifestation of a systemic autoimmune disease. Of these, 35–60% are IgG4‐related. Secondary RPF is linked to multiple risk factors, such as medications, malignancies, infection, radiation therapies and surgeries.^1^

Currently, there is no consensus on the optimal management of this low‐incidence disease. First‐line management is characterized by decompression of ureteral obstruction with indwelling double‐J stents or nephrostomy tubes, followed by medical therapy. When medical management fails, or chronic stenting is not desired or possible, ureterolysis is performed. The ureter is displaced from the fibrotic area using intraperitoneal transposition, with either a peritoneal flap or omental wrap. Historically, it has been performed with an open approach through a midline incision, but recently, laparoscopic and multiport robot‐assisted ureterolysis have been described.^2^, ^3^, ^4^, ^5^, ^6^, ^7^, ^8^ Although it achieves a high technical success rate in specialized centres, it is associated with high morbidity or the need for secondary procedures.

We believe that there are several advantages, including short recovery time, improved cosmesis and reduced postoperative pain, to performing a ureterolysis with single‐port (SP) and have begun to perform all our cases in this manner. Indeed, the SP platform has demonstrated significant versatility in upper urinary tract surgery,^9^, ^10^, ^11^ allowing surgeons to tailor access strategies to the individual patient's anatomy and surgical history.^12^ We present data on a first series of patients undergoing SP robot‐assisted ureterolysis (SP‐RAU) for RPF.

## 2 PATIENTS AND METHODS

### 2.1 Patients and data set

Data for all consecutive patients who underwent SP‐RAU for RPF between April 2016 and June 2024 were prospectively collected in our institutional review board‐approved dataset. All patients underwent CT; additionally, anatomical and functional evaluation was performed with pyelography in six cases (29%) and renal dynamic scintigraphy in three cases (14%). No preoperative biopsies were performed.

Patients with at least 12 months of follow‐up were included. Hydronephrosis was reported according to Onen's grading system,^13^ whereas complications were recorded following the Clavien Dindo classification.^14^ Bilateral cases were treated using a staged surgical strategy, with the contralateral side addressed in a separate surgical session. The primary endpoint was surgical success, defined as achievement of a stent‐free status at 12 months for each completed renal unit. Postoperatively, the initial trial of stent removal was scheduled at 4–6 weeks, preceded by retrograde pyelography to confirm patency. In cases of persistent obstruction, the stent was replaced; subsequent removal attempts were individualized, with routine stent exchanges performed at 3‐ or 6‐month intervals leading up to the 12‐month endpoint evaluation. Secondary endpoints included complete symptoms resolution, operative outcomes, complication rates and length of hospital stay. All procedures were performed at a single academic tertiary centre by a single expert surgeon using the da Vinci SP robotic system (Intuitive Surgical, Sunnyvale, CA, United States) under general anaesthesia using a propofol‐based total intravenous anaesthesia protocol. A multimodal analgesic strategy was employed to minimize opioid consumption, utilizing ultrasound‐guided regional nerve blocks and local anaesthetic infiltration at the surgical site. Postoperative care adhered to Enhanced Recovery After Surgery (ERAS) principles, emphasizing early mobilization and prompt oral intake. Readiness for same‐day discharge was strictly evaluated using objective criteria, requiring a Modified Aldrete Score of ≥9 to progress from early recovery and a Post‐Anaesthesia Discharge Scoring System (PADSS) score of ≥8 prior to home discharge.

### 2.2 Statistic

Statistical analysis was performed and reported following established guidelines.^15^ Descriptive statistics were generated using frequencies and proportions for categorical variables and medians with interquartile ranges (IQR) for continuous variables. Given the small sample size, comparisons between groups were performed using the non‐parametric Wilcoxon rank sum test, with effect sizes calculated using Cliff's delta and corresponding 95% confidence intervals (CI). The Hodges–Lehmann estimator was applied to provide median differences with confidence intervals. A two‐sided *p* value of <0.05 was considered statistically significant. All analyses were conducted with R software, Version 4.3.2 (R Foundation for Statistical Computing, Vienna, Austria).

## 3 RESULTS

### 3.1 Baseline characteristics

**FIGURE 1:**
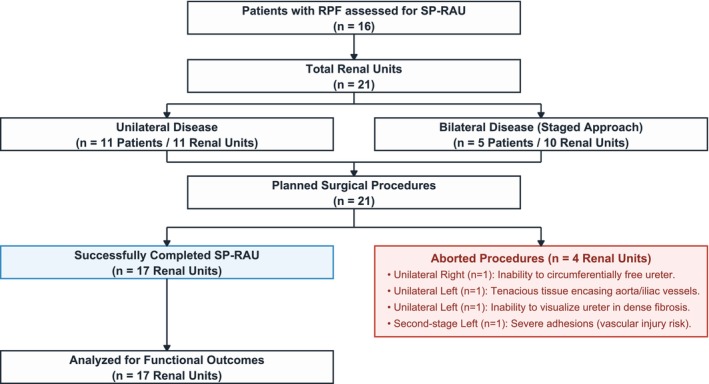
Study flowchart detailing patient selection and surgical outcomes for single‐port robot‐assisted ureterolysis (SP‐RAU).

Complete demographics are presented in Table 1. Overall, 16 patients accounting for 21 renal units underwent SP‐RAU (Figure 1). Eleven patients (69%) presented with unilateral disease (11 renal units), whereas five (31%) had bilateral disease (10 renal units). The median age was 50 years (IQR: 42–60), and five patients (31%) were female. The median body mass index (BMI) was 28 kg/m^2^ (IQR: 24–33). The median Charlson Comorbidity Index (CCI) was 3 (IQR: 2–4), with hypercholesterolemia and hypertension each observed in 31% of cases, diabetes and chronic obstructive pulmonary disease in 18% each and obesity in 37%. The median American Society of Anaesthesiologists (ASA) score was 3 (IQR: 2–3), and seven patients (43%) had previous abdominal surgery. Preoperative median serum creatinine (SC) was 2.2 mg/dl (IQR: 1.5–3), and the median estimated glomerular filtration rate (eGFR) was 29 ml/min/1.73 m^2^ (IQR: 21–51), with a median chronic kidney disease (CKD) stage of 3 (IQR: 1–4).

**TABLE 1:** Baseline characteristics of patients treated with single‐port robot‐assisted ureterolysis for retroperitoneal fibrosis.

Parameter	Patients (*n* = 16)
Median age at surgery, year (IQR)	50.5 (42–60.5)
Female, *n* (%)	5 (31.2)
Renal unit, *n* (%)	21
Median BMI, kg/m^2^ (IQR)	28.8 (24.7–33.3)
Smoking status, *n* (%)	
Never	7 (43.7)
Current	3 (18.8)
Smoker	6 (37.5)
Median age adjusted CCI (IQR)	3 (2–4)
Hypertension, *n* (%)	5 (31.2)
Hypercholesterolemia, *n* (%)	5 (31.2)
COPD, *n* (%)	3 (18.8)
Diabetes, *n* (%)	3 (18.8)
Obesity, *n* (%)	6 (37.5)
Median ASA score (IQR)	3 (2–3)
Previous abdominal surgery, *n* (%)	7 (43.8)
Median CKD stage (IQR)	3 (1–4)
Median preSC, mg/dl (IQR)	2.28 (1.51–3)
Median pre‐eGFR, ml/min/1.73 m^2^ (IQR)	29 (21–51)
Etiology, *n* (%)	
Primary	6 (37.5)
Secondary	10 (62.5)
Radiotherapy	5 (50)
Previous surgery	4 (40)
Malignancy	1 (10)
Hydronephrosis grade, *n* (%)	
2	4 (25)
3	11 (68.8)
4	1 (6.2)
Side, *n* (%)	
Left	9 (42.8)
Right	12 (57.2)
Symptoms, *n* (%)	
Flank pain	17 (80.9)
Urinary tract infection	1 (4.8)
Hematuria	3 (14.3)
Type of PTUD, *n* (%)	
Double‐J stent	18 (85.7)
Nephrostomy	1 (4.8)
Double‐J stent and nephrostomy	2 (9.5)
Median Stent duration, months (IQR)	11.5 (5.5–35.3)

Abbreviations: ASA, American Society of Anesthesiologists; BMI, body mass index; CCI, Charlson comorbidity index; CKD, chronic kidney disease; COPD, chronic obstructive pulmonary disease; DNRS, dynamic nuclear renal scan; IQR, interquartile range; LAA, low anterior access; pre‐eGFR, median preoperative estimated glomerular filtration rate; prSC, preoperative serum creatinine; PTUD, preoperative temporary urinary diversion.

RPF was idiopathic in 37% of cases and secondary in 62%; these were mainly from previous radiotherapy 50% and prior surgery 40%. Prior surgical procedures included open repair of an abdominal aortic aneurysm, radical hysterectomy with pelvic and para‐aortic lymph node dissection, left pyeloplasty and a caesarean section. The hydronephrosis was Grade 2 in 25%, Grade 3 in 68% and Grade 4 in 6%. The most frequent symptoms were flank pain in 80%, followed by hematuria in 14% and recurrent urinary tract infection in 4.8%. Before the procedure, 85% of patients had a double‐J stent in place, with a median stent duration of 11 month (IQR: 5.5–35).

### 3.2 Aborted procedures

Four procedures (19%) were aborted: In the unilateral cohort, three procedures were halted, one on the right side due to an inability to circumferentially free the ureter and two on the left side, one due to tenacious tissue encasing the aorta and iliac vessels and another due to an inability to visualize the ureter within dense fibrosis. In the bilateral cohort, one second‐stage left ureterolysis was aborted due to severe adhesions involving the aorta and iliac vessels, which posed an unacceptably elevated risk of vascular injury (Video S1). These procedures were excluded from intraoperative and postoperative analyses.

Notably, median preoperative stent duration was significantly longer in patients with aborted procedures compared with completed ones: 60 months (IQR: 47–128) versus 9 months (IQR: 4–29; *p* = 0.006). The Hodges–Lehmann estimator showed a median difference of 51 months (95% CI: 8–189), with a large effect size (Cliff's delta = 0.86, 95% CI: 0.53–1).

### 3.3 Intraoperative outcomes

Seventeen renal units were treated and included in the intraoperative analysis (Table 2). The median operative time was 215 min (IQR: 195–240), and the median blood loss was 10 ml (IQR: 10–20). No intraoperative transfusion, conversions to open procedure or complications occurred. A transperitoneal approach was used in 94%, whereas retroperitoneal access was performed in one patient (5.9%), with patient positioning distributed between the lateral flank (53%) and the supine position (47%). Intraperitonealization was performed in 15 renal units (88%), peritoneal flap in 11 (64%) and omental wrap in 3 (17%). The choice between a peritoneal flap and an omental wrap was dictated by intraoperative tissue availability; an omental wrap was preferred when sufficient mobile omentum could reach the ureter without tension, whereas a local peritoneal flap was harvested in all other instances to ensure adequate ureteral isolation from the fibrotic retroperitoneum.

**TABLE 2:** Intraoperative and early postoperative outcomes per surgical procedure of single‐port robot‐assisted ureterolysis for retroperitoneal fibrosis.

Characteristic	Renal units (*n* = 17)
Median Operative Time, minutes (IQR)	215 (195–240)
Median EBL, cm^3^ (IQR)	10 (10–20)
Position, *n* (%)	
Flank	9 (52.9)
Supine	8 (47.1)
Approach, *n* (%)	
Transperitoneal	16 (94.1)
Retroperitoneal	1 (5.9)
Surgical procedure, *n* (%)	
Intraperitonealization	15 (88.2)
Peritoneal flap	11 (64.7)
Omental wrap	3 (17.6)
Additional procedure, *n* (%)	5 (29.4)
Transfusion, *n* (%)	0
Intraoperative complication, *n* (%)	0
Open conversion, *n* (%)	0
Drain placement, *n* (%)	4 (23.5)
Ureteral stent placement, *n* (%)	17 (100)
Median LOS, hours (IQR)	11.5 (7.8–26.2)
Same‐day discharge, *n* (%)	9 (52.9)
Opioid use during hospitalization, *n* (%)	13 (76.5)
Opioid prescribed at discharge, *n* (%)	2 (11.8)

Abbreviations: EBL, estimated blood loss; IQR, interquartile range; LOS, length of stay; OT, operative time; PTUD, preoperative temporary urinary diversion; TPA, transperitoneal access.

Additional reconstructive manoeuvres were needed in five cases (29%), including two ureteroplasties with buccal mucosal graft, one ipsilateral pyeloplasty, one ureteral reimplantation for extensive fibrosis and one repair of two preexisting small ureterotomies exposing the indwelling ureteral stent. None of these reconstructive manoeuvres were planned preoperatively; rather, they became necessary based on intraoperative findings. Four procedures required drain placement (23%).

Median length of hospital stay (LOS) was 11 h (IQR: 7.8–26), with a same‐day discharge (SDD) rate of 52%. Opioids were administered during hospitalization in 13 (76%) patients but prescribed at discharge in only two (11%).

### 3.4 Postoperative outcomes

Postoperative outcomes are summarized in Table 3. Median follow‐up was 25 months (IQR: 18–50). Complete symptom resolution was reported in 14 renal units (82%) at follow up. Hydronephrosis improved in eight (47%), remained stable in eight (47%) and worsened in one (5.9%). Stent‐free status was achieved in 11 renal units (64%) at 12 months. At follow‐up, median ΔeGFR was +6 ml/min/1.73m^2^ (95% CI: −5–14, *p* = 0.46), with 8 of 13 patients (61%) showing improved renal function.

**TABLE 3:** Postoperative outcomes per surgical procedure of single‐port robot‐assisted ureterolysis for retroperitoneal fibrosis.

Characteristic	Renal units (*n* = 17)
Postoperative Complications, *n* (%)	1 (5.9)
Clavien Dindo II	1 (100)
Median FU, months (IQR)	25 (18–50)
Symptom resolution, *n* (%)	14 (82)
Hydronephrosis, *n* (%)	
Stable	8 (47)
Improved	8 (47)
Worsened	1 (5.9)
Stent‐free, *n* (%)	
3 months	6 (35)
12 months	11 (64)
Nephrostomy‐free, *n* (%)	
3 months	17 (100)
12 months	17 (100)
Median postSC, mg/dl (IQR)^a^	1.69 (1.4–2.7)
Median post‐eGFR, ml/min/1.73 m^2^ (IQR)^a^	48 (28–55)
Median Δ eGFR, ml/min/1.73 m^2^ (IQR)^a^	6 (−5 to 14)

Abbreviations: eGFR, estimated glomerular filtration rate; FU, follow‐up; post‐eGFR, median postoperative estimated glomerular filtration rate; postSC, postoperative serum creatinine; RoH, resolution of hydronephrosis; TSR, time to stent removal; UO, urinary obstruction.

^a^ Systemic functional outcomes were assessed per patient (*n* = 13).

## 4 DISCUSSION

SP‐RAU is a novel approach in the minimally invasive management of ureteral obstruction induced by RPF. To the best of our knowledge, our study is the first series reporting SP‐RAU.

Open ureterolysis series have reported success rates of approximately 90%, but at the cost of considerable morbidity, with postoperative complication rates reaching up to 26%, median blood loss of 390 ml and a median hospitalization exceeding 1 week.^2^, ^4^ Laparoscopic ureterolysis subsequently emerged as a less morbid alternative. Fugita et al.^3^ reported that 92% of patients remained obstruction‐free at 30 months, although the procedure remained technically demanding and was associated with a 30% postoperative complication rate. Keehn et al.,^12^ in a series of multiport robot‐assisted ureterolysis, reported a mean blood loss of 51 ml, a mean hospital stay of 2.6 days and 100% relief of obstruction, with 14.3% of patients achieving this outcome only after a secondary procedure.

Compared with these historical series, our SP‐RAU cohort demonstrated comparable efficacy, with 64% of renal units stent‐free at 12 months without requiring secondary procedures and 82% reporting complete symptom resolution. We specifically selected stent‐free status at 12 months as our primary endpoint because it reflects the ultimate clinical goal of ureterolysis: freeing the patient from the burden of long‐term stenting. These results were achieved with a markedly shorter median length of stay of 11 h and minimal blood loss of 10 ml.

In our experience, same‐day discharge was feasible in over half of patients, with minimal opioid requirements, mirroring recovery profiles reported in other single‐port reconstructive procedure.^16^ Importantly, SP‐RAU was successful even in patients with prior abdominal surgery and complex secondary fibrosis, highlighting the flexibility of the SP platform, which can be performed reducing perioperative morbidity and enhancing rapid recovery.^17^

Regarding the choice of surgical approach, the transperitoneal route was employed in the vast majority of our cases. This was dictated by the requirements of the procedure itself: ureterolysis for RPF frequently necessitates reconstructive manoeuvres such as omental wrapping, peritoneal flap coverage and ureteral intraperitonealization, all of which require direct access to the peritoneal cavity. These steps are considered essential to prevent fibrotic recurrence and new encasement of the freed ureter. A retroperitoneal approach, while increasingly adopted for SP renal surgery, with emerging consensus on its standardization,^18^ does not provide adequate exposure of the omentum or peritoneal surfaces. In our series, the retroperitoneal route was reserved for a single case in which patient anatomy and surgical history favoured a direct posterior approach without the need for ureteral wrapping.

Nevertheless, 19% of procedures were aborted due to severe fibrosis or unacceptable vascular risk. These cases underscore the importance of surgical judgement. As shown by our supplementary video, the ability to recognize when to stop dissection is a critical safety part in the management of RPF, and surgeons must be prepared to terminate the procedure when safe mobilization of the ureter cannot be achieved, rather than risk major complications. Notably, patients with aborted procedures had significantly longer preoperative stent duration (60 vs. 9 months, *p* = 0.006). To our knowledge, this is the first report suggesting a link between prolonged indwelling stents and increased operative complexity. Chronic indwelling stents may perpetuate inflammation, promote ureteral adherence to fibrotic tissue or obscure tissue planes, thereby elevating procedural risk. These observations highlight the importance of timely surgical referral, rather than delaying definitive intervention. A history of prolonged stenting should be carefully weighed during patient selection and preoperative counselling, as it may significantly increase the likelihood of an aborted procedure. Furthermore, given that nearly 30% of completed cases in our series required unplanned reconstructive manoeuvres due to ischemic or poor‐quality ureteral tissue, comprehensive preoperative counselling is mandatory. Informed consent must explicitly include the potential need for complex, additional surgical outcomes, such as buccal mucosal grafts, pyeloplasty or ureteral reimplantation. Assessing true renal functional recovery in RPF remains complex: Although we observed a trend towards eGFR stabilization, it is heavily confounded by the duration of the obstruction and preexisting irreversible parenchymal damage. Additionally, the prevalence of secondary RPF and systemic comorbidities in our cohort makes it difficult to isolate the exact impact of ureterolysis. In this heavily pre‐treated population, the stabilization of renal function should often be viewed as a clinical success.

Our study is strengthened by multiple factors: prospective data collection, standardized technique and adequate follow‐up duration. Several limitations should also be acknowledged. First, the small sample size limits statistical power and generalizability. Second, the single‐surgeon, single‐centre design may not reflect outcomes across different surgical teams or institutional settings. Nevertheless, recent comprehensive evaluations of the single‐port landscape confirm the safety, versatility and reproducibility of this platform across a wide array of standardized urological procedures.^10^ Third, we did not include a comparator arm precluding a direct assessment of superiority against multiport, laparoscopic or open procedures. Furthermore, postoperative evaluation of surgical success primarily relied on clinical symptom resolution, renal function trends and morphological imaging, without the routine use of diuretic renography. Lastly, although functional outcomes were encouraging, longer follow‐up is needed to assess the risk of late recurrence.

## 5 CONCLUSIONS

SP‐RAU is a safe and feasible minimally invasive option for the surgical management of ureteral obstruction secondary to RPF. In our experience, the technique achieved high rates of symptom resolution and satisfactory functional outcomes, while markedly reducing hospitalization time and perioperative morbidity compared with traditional approaches. Importantly, the feasibility of same‐day discharge in over half of the patients further underscores its potential to optimize recovery and resource utilization.

Nonetheless, nearly one in five procedures was aborted, emphasizing the critical importance of intraoperative judgement. The observed association between prolonged preoperative stenting and increased operative complexity warrants further investigation and may have implications for surgical timing and preoperative planning.

Future multicentre comparative studies with extended follow‐up are needed to validate these findings, refine patient selection criteria and better define the role of SP‐RAU relative to multiport robotic, laparoscopic and open ureterolysis.

## AUTHOR CONTRIBUTIONS

*Conception and design*: Filippo Carletti and Simone Crivellaro. *Data acquisition*: Filippo Carletti, Flavia Tamborino and Alexandru Turcan. *Critical revision for scientific and factual content*: Filippo Carletti, Flavia Tamborino, Alexandru Turcan, Lorenzo Salvodirocco, Luca Alfredo Morgantini and Marwan Alkassis. *Drafting the manuscript*: Filippo Carletti, Flavia Tamborino, Alexandru Turcan and Marwan Alkassis. *Statistical analysis*: Filippo Carletti. *Supervision*: Simone Crivellaro and Fabrizio Dal Moro. *Patient selection and data*: Filippo Carletti, Flavia Tamborino and Alexandru Turcan. *Review and revisions*: Filippo Carletti, Flavia Tamborino, Alexandru Turcan, Luca Alfredo Morgantini and Marwan Alkassis. *Data interpretation*: Filippo Carletti, Alexandru Turcan, Flavia Tamborino and Simone Crivellaro.

## CONFLICT OF INTEREST STATEMENT

Simone Crivellaro is a consultant for Intuitive Surgical, Inc. All other authors have nothing to disclose.

## Supporting information


**Video S1.** Supporting Information.
